# Tetra­butyl­ammonium tetra­kis­(trimethyl­silanolato-κ*O*)ferrate(III)

**DOI:** 10.1107/S1600536812035337

**Published:** 2012-08-23

**Authors:** Michael Hay, Richard Staples, Andre Lee

**Affiliations:** a Penn State Beaver, 100 University Drive, Monaca, PA 15061, USA; b Center for Crystallographic Research, Michigan State University, Department of Chemistry East, Lansing, MI 48824, USA; c Michigan State University, Chemical Engineering and Material Science, 3514 Engineering Building, East Lansing, MI 48824, USA

## Abstract

In the title salt, (C_16_H_36_N)[Fe(C_3_H_9_OSi)_4_], the cation contains a central N atom bonded to four *n*-butyl alkyl groups in a tetra­hedral arrangement, while the anion contains a central Fe^III^ atom tetra­hedrally coordinated by four trimethyl­silanolate ligands.

## Related literature

For general background to the structural characterization of silsesquioxane compounds containing tetra­butyl­ammonium iron(III), see: Hay & Geib (2007 ▶); Hay *et al.* (2003 ▶, 2009 ▶). For details of the synthesis, see: Shapley *et al.* (2003 ▶).
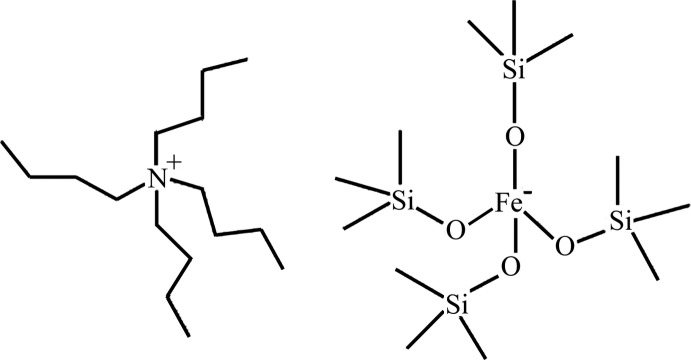


## Experimental

### 

#### Crystal data

(C_16_H_36_N)[Fe(C_3_H_9_OSi)_4_]
*M* *_r_* = 655.08
Triclinic, 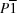

*a* = 10.4952 (5) Å
*b* = 10.5143 (5) Å
*c* = 19.3506 (9) Å
α = 82.722 (1)°
β = 82.834 (1)°
γ = 81.658 (1)°
*V* = 2083.37 (17) Å^3^
*Z* = 2
Mo *K*α radiation
μ = 0.50 mm^−1^
*T* = 173 K
0.56 × 0.35 × 0.28 mm

#### Data collection

Bruker APEXII CCD diffractometer
Absorption correction: multi-scan (*SADABS*; Bruker, 2008 ▶) *T*_min_ = 0.767, *T*_max_ = 0.872
18068 measured reflections
9424 independent reflections
6223 reflections with *I* > 2σ(*I*)
*R*_int_ = 0.071

#### Refinement

*R*[*F* ^2^ > 2σ(*F* ^2^)] = 0.041
*wR*(*F* ^2^) = 0.088
*S* = 0.93
9424 reflections
359 parameters
H-atom parameters constrained
Δρ_max_ = 0.36 e Å^−3^
Δρ_min_ = −0.30 e Å^−3^

### 

Data collection: *APEX2* (Bruker, 2008 ▶); cell refinement: *SAINT* (Bruker, 2008 ▶); data reduction: *SAINT*; program(s) used to solve structure: *SHELXS97* (Sheldrick, 2008 ▶); program(s) used to refine structure: *SHELXL97* (Sheldrick, 2008 ▶); molecular graphics: *SHELXTL* (Sheldrick, 2008 ▶); software used to prepare material for publication: *SHELXTL*.

## Supplementary Material

Crystal structure: contains datablock(s) I, global. DOI: 10.1107/S1600536812035337/vn2048sup1.cif

Structure factors: contains datablock(s) I. DOI: 10.1107/S1600536812035337/vn2048Isup2.hkl

Additional supplementary materials:  crystallographic information; 3D view; checkCIF report

## Figures and Tables

**Table 1:** Selected bond lengths (Å)

Fe1—O1	1.8608 (13)
Fe1—O2	1.8591 (13)
Fe1—O3	1.8515 (14)
Fe1—O4	1.8583 (13)

**Fig. 1.**
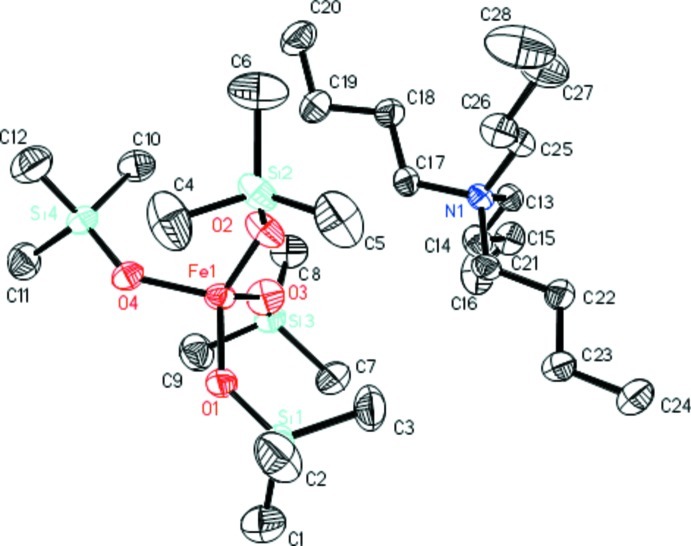
A 50% thermal ellipsoidal drawing of the asymmetric cell.

**Fig. 2.**
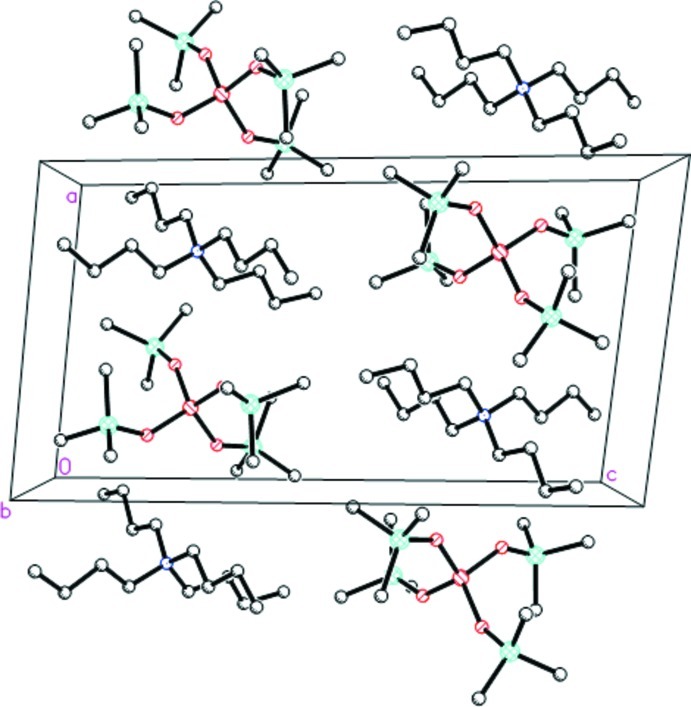
Drawing of the packing along the b axis.

## supplementary crystallographic information

### Comment

Over the past decade, Hay *et al.* reported on the structural characterization of numerous tetrabutylammonium iron (III) containing silsesquioxane compounds (Hay *et al.*, 2003; Hay & Geib, 2007, Hay *et al.*, 2009). In order to make a more complete structural study of these compounds, it was useful to have structural data on an analogous tetrabutylammonium iron (III) silanolato compound – the title compound (Fig. 1). Its structural arrangement contains a pair of tetrabutylammonium cations and tetrakistrimethylsilanolato ferrate (III) anions in a triclinic unit cell (Fig. 2). The tetrabutylammonium cation, C_16_H_36_N^+^, consists of a tetrahedrally arranged central nitrogen atom, with N—C bond lengths in the range of 1.515 (2)–1.520 (2) Å and C—N—C bond angles in the range of 105.74 (13)–111.41 (14)°. The complex anion, C_12_H_36_FeO_4_Si_4_^-^, contains a four coordinate Fe^III^ atom with a tetrahedral arrangement of four trimethylsilanolato ligands. The O—Fe—O bond angles are 105.17 (6)–112.58 (6)°. The Fe—O bond lengths are in the range of 1.8515 (14)–1.8608 (13) Å.

### Experimental

The following synthetic protocol is adapted from the previously reported procedure (Shapley, *et al.*, 2003). A yellow solution of [C_16_H_36_N][FeCl_4_] (1.136 mmol, 0.5000 g) in dichloromethane (10 ml) was treated with four equivalents of solid sodium trimethlysilanate (4.544 mmol, 0.5098 g). Immediately, the yellow solution turned red due to the formation of a dark red precipitate. The reaction mixture was stirred for 40 minutes before the precipitate was removed by filtration through celite. The resulting pale green filtrate was concentrated under reduced pressure to give a pale green powder. The powder was extracted with diethyl ether and filtered to remove any insoluble material. Hexanes were added to the diethyl ether filtrate and the sample was stored at 243 K for about 30 minutes before colorless block crystals formed which were analyzed.

### Refinement

All H atoms were placed in calculated positions and refined using a riding model. C—H(aromatic) = 0.94 Å and *U*_iso_(H) = 1.2*U*_eq_(C); C—H (alaphatic) = 0.99 Å and *U*_iso_(H) = 1.2*U*_eq_(C); CH_2_ = 0.98 Å and *U*_iso_(H) = 1.2*U*_eq_(C); CH_3_ = 0.97 Å and *U*_iso_(H) = 1.5*U*_eq_(C); N—H = 0.86 (0.92) Å and *U*_iso_(H) = 1.2*U*_eq_(N); O—H(alcohol) = 0.85 Å and *U*_iso_(H) = 1.2*U*_eq_(O); O—H(acid) = 0.82 Å and *U*_iso_(H) = 1.5*U*_eq_(O). ?

### Crystal data

**Table:**

(C_16_H_36_N)[Fe(C_3_H_9_OSi)_4_]	*Z* = 2
*M**_r_* = 655.08	*F*(000) = 722
Triclinic, *P*1	*D*_x_ = 1.044 Mg m^−^^3^
Hall symbol: -P 1	Mo *K*α radiation, λ = 0.71073 Å
*a* = 10.4952 (5) Å	Cell parameters from 7900 reflections
*b* = 10.5143 (5) Å	θ = 2.3–27.4°
*c* = 19.3506 (9) Å	µ = 0.50 mm^−^^1^
α = 82.722 (1)°	*T* = 173 K
β = 82.834 (1)°	Block, colourless
γ = 81.658 (1)°	0.56 × 0.35 × 0.28 mm
*V* = 2083.37 (17) Å^3^	

### Data collection

**Table:**

Bruker APEXII CCD diffractometer	9424 independent reflections
Radiation source: fine-focus sealed tube	6223 reflections with *I* > 2σ(*I*)
Graphite monochromator	*R*_int_ = 0.071
Detector resolution: 836.6 pixels mm^-1^	θ_max_ = 28.6°, θ_min_ = 2.0°
ω and φ 0.5 deg scans	*h* = −14→13
Absorption correction: multi-scan (*SADABS*; Bruker, 2008)	*k* = −13→14
*T*_min_ = 0.767, *T*_max_ = 0.872	*l* = −25→25
18068 measured reflections	

### Refinement

**Table:**

Refinement on *F*^2^	Primary atom site location: structure-invariant direct methods
Least-squares matrix: full	Secondary atom site location: difference Fourier map
*R*[*F*^2^ > 2σ(*F*^2^)] = 0.041	Hydrogen site location: inferred from neighbouring sites
*wR*(*F*^2^) = 0.088	H-atom parameters constrained
*S* = 0.93	*w* = 1/[σ^2^(*F*_o_^2^) + (0.0118*P*)^2^] where *P* = (*F*_o_^2^ + 2*F*_c_^2^)/3
9424 reflections	(Δ/σ)_max_ = 0.002
359 parameters	Δρ_max_ = 0.36 e Å^−^^3^
0 restraints	Δρ_min_ = −0.30 e Å^−^^3^

### Special details

**Table:**

Experimental. Data was collected using a BRUKER CCD (charge coupled device) based diffractometer equipped with an Oxford low-temperature apparatus operating at 173 K. A suitable crystal was chosen and mounted on a glass fiber or nylon loop using Paratone oil for Mo radiation and Mineral oil for Copper radiation. Data were measured using omega and phi scans of 0.5° per frame for 30 s. The total number of images were based on results from the program *COSMO* where redundancy was expected to be 4 and completeness to 0.83 Å to 100%. Cell parameters were retrieved using *APEX* II software and refined using *SAINT* on all observed reflections. Data reduction was performed using the *SAINT* software which corrects for Lp. Scaling and absorption corrections were applied using *SADABS6* multi-scan technique (Sheldrick, 2008). The structures are solved by the direct method using the *SHELXS97* program and refined by least squares method on *F*^2^, *SHELXL97*, incorporated in *SHELXTL*-PC.
Geometry. All e.s.d.'s (except the e.s.d. in the dihedral angle between two l.s. planes) are estimated using the full covariance matrix. The cell e.s.d.'s are taken into account individually in the estimation of e.s.d.'s in distances, angles and torsion angles; correlations between e.s.d.'s in cell parameters are only used when they are defined by crystal symmetry. An approximate (isotropic) treatment of cell e.s.d.'s is used for estimating e.s.d.'s involving l.s. planes.
Refinement. _michigan_contact_Crystallographer_name 'Richard Staples' _michigan_contact_Crystallographer_email xraystaples@chemistry.msu.com

### Fractional atomic coordinates and isotropic or equivalent isotropic displacement parameters (Å^2^)

**Table:**

	*x*	*y*	*z*	*U*_iso_*/*U*_eq_	
Fe1	0.74285 (3)	0.64074 (3)	0.746004 (14)	0.03091 (9)	
Si1	0.79239 (6)	0.42170 (6)	0.87610 (3)	0.04012 (16)	
Si2	0.72382 (6)	0.43686 (6)	0.63710 (3)	0.04463 (17)	
Si3	0.54754 (6)	0.86616 (6)	0.82993 (3)	0.03772 (15)	
Si4	0.88113 (6)	0.84989 (6)	0.63377 (3)	0.03860 (16)	
O1	0.82805 (13)	0.52860 (13)	0.81292 (7)	0.0461 (4)	
O2	0.67258 (13)	0.54525 (13)	0.68921 (7)	0.0456 (4)	
O3	0.60452 (14)	0.74786 (13)	0.78552 (7)	0.0483 (4)	
O4	0.86943 (13)	0.73219 (13)	0.69549 (7)	0.0416 (4)	
C1	0.8425 (3)	0.4626 (3)	0.95884 (12)	0.0724 (8)	
H1A	0.9368	0.4608	0.9541	0.109*	
H1B	0.8164	0.3994	0.9978	0.109*	
H1C	0.8007	0.5493	0.9681	0.109*	
C2	0.8795 (2)	0.2593 (2)	0.85816 (15)	0.0737 (9)	
H2A	0.8566	0.2371	0.8140	0.111*	
H2B	0.8546	0.1942	0.8965	0.111*	
H2C	0.9732	0.2612	0.8546	0.111*	
C3	0.6159 (2)	0.4104 (3)	0.88869 (14)	0.0755 (9)	
H3A	0.5684	0.4925	0.9020	0.113*	
H3B	0.5979	0.3407	0.9258	0.113*	
H3C	0.5881	0.3919	0.8449	0.113*	
C4	0.9026 (2)	0.4104 (3)	0.62274 (16)	0.0805 (9)	
H4A	0.9343	0.4884	0.5974	0.121*	
H4B	0.9306	0.3374	0.5951	0.121*	
H4C	0.9377	0.3913	0.6681	0.121*	
C5	0.6691 (2)	0.2787 (2)	0.67500 (16)	0.0762 (9)	
H5A	0.7098	0.2473	0.7179	0.114*	
H5B	0.6941	0.2155	0.6410	0.114*	
H5C	0.5747	0.2903	0.6859	0.114*	
C6	0.6578 (3)	0.4858 (3)	0.55180 (13)	0.0842 (9)	
H6A	0.5632	0.4910	0.5587	0.126*	
H6B	0.6930	0.4217	0.5194	0.126*	
H6C	0.6824	0.5705	0.5322	0.126*	
C7	0.4713 (2)	0.8052 (2)	0.91776 (11)	0.0580 (7)	
H7A	0.3996	0.7586	0.9120	0.087*	
H7B	0.4384	0.8783	0.9446	0.087*	
H7C	0.5360	0.7465	0.9428	0.087*	
C8	0.4215 (2)	0.9769 (2)	0.78437 (12)	0.0525 (6)	
H8A	0.4607	1.0150	0.7393	0.079*	
H8B	0.3846	1.0459	0.8135	0.079*	
H8C	0.3528	0.9282	0.7764	0.079*	
C9	0.6750 (2)	0.9638 (2)	0.84233 (13)	0.0593 (7)	
H9A	0.7423	0.9088	0.8672	0.089*	
H9B	0.6366	1.0346	0.8699	0.089*	
H9C	0.7134	0.9996	0.7965	0.089*	
C10	0.7182 (2)	0.9279 (2)	0.61219 (12)	0.0544 (7)	
H10A	0.6715	0.8637	0.5972	0.082*	
H10B	0.7277	0.9982	0.5743	0.082*	
H10C	0.6695	0.9629	0.6537	0.082*	
C11	0.9695 (2)	0.9731 (2)	0.66132 (12)	0.0529 (6)	
H11A	0.9220	1.0071	0.7034	0.079*	
H11B	0.9766	1.0440	0.6235	0.079*	
H11C	1.0564	0.9331	0.6715	0.079*	
C12	0.9728 (2)	0.7892 (2)	0.55258 (12)	0.0647 (7)	
H12A	1.0562	0.7410	0.5638	0.097*	
H12B	0.9878	0.8627	0.5174	0.097*	
H12C	0.9224	0.7322	0.5341	0.097*	
N1	0.24290 (14)	0.52480 (15)	0.75675 (8)	0.0290 (4)	
C13	0.12540 (17)	0.61155 (18)	0.78604 (10)	0.0324 (5)	
H13A	0.0806	0.6579	0.7462	0.039*	
H13B	0.0651	0.5559	0.8142	0.039*	
C14	0.15129 (19)	0.7106 (2)	0.83111 (11)	0.0417 (5)	
H14A	0.1976	0.6660	0.8707	0.050*	
H14B	0.2078	0.7701	0.8030	0.050*	
C15	0.0275 (2)	0.7877 (2)	0.85923 (11)	0.0438 (6)	
H15A	−0.0253	0.7287	0.8906	0.053*	
H15B	−0.0224	0.8250	0.8196	0.053*	
C16	0.0493 (2)	0.8961 (2)	0.89928 (14)	0.0655 (7)	
H16A	0.0958	0.8598	0.9397	0.098*	
H16B	−0.0345	0.9430	0.9156	0.098*	
H16C	0.1007	0.9556	0.8685	0.098*	
C17	0.34387 (18)	0.60341 (19)	0.71623 (10)	0.0326 (5)	
H17A	0.4220	0.5432	0.7025	0.039*	
H17B	0.3688	0.6603	0.7479	0.039*	
C18	0.30222 (19)	0.6867 (2)	0.65075 (10)	0.0391 (5)	
H18A	0.2888	0.6304	0.6157	0.047*	
H18B	0.2189	0.7409	0.6627	0.047*	
C19	0.4030 (2)	0.7727 (2)	0.61963 (11)	0.0444 (6)	
H19A	0.4883	0.7191	0.6125	0.053*	
H19B	0.4098	0.8349	0.6530	0.053*	
C20	0.3703 (2)	0.8464 (2)	0.55050 (11)	0.0601 (7)	
H20A	0.3713	0.7853	0.5161	0.090*	
H20B	0.4345	0.9055	0.5337	0.090*	
H20C	0.2840	0.8961	0.5568	0.090*	
C21	0.31012 (18)	0.44134 (18)	0.81477 (10)	0.0329 (5)	
H21A	0.3380	0.4990	0.8450	0.039*	
H21B	0.3893	0.3916	0.7932	0.039*	
C22	0.23097 (19)	0.3468 (2)	0.86101 (10)	0.0400 (5)	
H22A	0.1541	0.3951	0.8854	0.048*	
H22B	0.2004	0.2894	0.8316	0.048*	
C23	0.3114 (2)	0.2658 (2)	0.91458 (10)	0.0420 (5)	
H23A	0.3423	0.3236	0.9437	0.050*	
H23B	0.3882	0.2178	0.8899	0.050*	
C24	0.2344 (2)	0.1701 (2)	0.96193 (12)	0.0609 (7)	
H24A	0.1604	0.2175	0.9881	0.091*	
H24B	0.2902	0.1181	0.9949	0.091*	
H24C	0.2030	0.1132	0.9333	0.091*	
C25	0.19311 (19)	0.44218 (19)	0.70961 (10)	0.0344 (5)	
H25A	0.1265	0.3941	0.7378	0.041*	
H25B	0.1502	0.5003	0.6726	0.041*	
C26	0.2951 (2)	0.3457 (2)	0.67477 (11)	0.0448 (6)	
H26A	0.3357	0.2839	0.7112	0.054*	
H26B	0.3635	0.3922	0.6471	0.054*	
C27	0.2357 (2)	0.2720 (2)	0.62695 (13)	0.0622 (7)	
H27A	0.1715	0.2212	0.6554	0.075*	
H27B	0.1895	0.3346	0.5929	0.075*	
C28	0.3361 (3)	0.1820 (3)	0.58759 (15)	0.0907 (10)	
H28A	0.3972	0.2323	0.5574	0.136*	
H28B	0.2933	0.1345	0.5588	0.136*	
H28C	0.3830	0.1206	0.6211	0.136*	

### Atomic displacement parameters (Å^2^)

**Table:**

	*U*^11^	*U*^22^	*U*^33^	*U*^12^	*U*^13^	*U*^23^
Fe1	0.03597 (17)	0.02678 (16)	0.03030 (17)	−0.00261 (13)	−0.00944 (13)	−0.00036 (12)
Si1	0.0344 (3)	0.0395 (4)	0.0461 (4)	−0.0115 (3)	−0.0156 (3)	0.0149 (3)
Si2	0.0378 (4)	0.0445 (4)	0.0565 (4)	0.0014 (3)	−0.0152 (3)	−0.0219 (3)
Si3	0.0411 (4)	0.0376 (3)	0.0337 (3)	−0.0011 (3)	−0.0025 (3)	−0.0072 (3)
Si4	0.0440 (4)	0.0387 (3)	0.0314 (3)	−0.0072 (3)	−0.0058 (3)	0.0063 (3)
O1	0.0402 (8)	0.0498 (9)	0.0476 (9)	−0.0145 (7)	−0.0188 (7)	0.0210 (7)
O2	0.0356 (8)	0.0480 (9)	0.0580 (10)	0.0021 (7)	−0.0150 (8)	−0.0235 (8)
O3	0.0533 (9)	0.0416 (9)	0.0477 (9)	0.0050 (8)	−0.0012 (8)	−0.0130 (7)
O4	0.0465 (9)	0.0388 (8)	0.0377 (8)	−0.0081 (7)	−0.0100 (7)	0.0114 (7)
C1	0.082 (2)	0.087 (2)	0.0508 (16)	−0.0161 (17)	−0.0132 (15)	−0.0040 (15)
C2	0.0672 (18)	0.0410 (15)	0.119 (2)	−0.0111 (14)	−0.0364 (18)	0.0005 (15)
C3	0.0402 (15)	0.083 (2)	0.097 (2)	−0.0209 (15)	−0.0119 (15)	0.0309 (17)
C4	0.0470 (16)	0.086 (2)	0.114 (2)	−0.0026 (16)	0.0061 (17)	−0.0519 (19)
C5	0.0668 (18)	0.0441 (15)	0.120 (3)	0.0008 (14)	−0.0237 (18)	−0.0148 (16)
C6	0.111 (2)	0.084 (2)	0.0618 (19)	0.0044 (19)	−0.0300 (18)	−0.0245 (16)
C7	0.0594 (16)	0.0718 (17)	0.0410 (14)	−0.0125 (14)	0.0010 (12)	−0.0021 (12)
C8	0.0564 (15)	0.0482 (14)	0.0511 (15)	0.0045 (12)	−0.0079 (12)	−0.0102 (12)
C9	0.0579 (16)	0.0612 (16)	0.0626 (17)	−0.0139 (14)	−0.0074 (14)	−0.0136 (13)
C10	0.0578 (15)	0.0532 (15)	0.0488 (15)	−0.0031 (13)	−0.0166 (13)	0.0130 (12)
C11	0.0542 (15)	0.0461 (14)	0.0578 (15)	−0.0126 (12)	−0.0058 (13)	0.0032 (12)
C12	0.0726 (18)	0.0747 (18)	0.0430 (15)	−0.0062 (15)	0.0006 (14)	−0.0025 (13)
N1	0.0224 (8)	0.0352 (9)	0.0299 (9)	−0.0038 (7)	−0.0066 (7)	−0.0014 (7)
C13	0.0224 (10)	0.0390 (12)	0.0350 (12)	−0.0030 (9)	−0.0043 (9)	−0.0013 (9)
C14	0.0330 (12)	0.0459 (13)	0.0467 (13)	−0.0041 (11)	−0.0023 (11)	−0.0106 (11)
C15	0.0413 (13)	0.0398 (13)	0.0477 (14)	−0.0014 (11)	0.0012 (11)	−0.0050 (11)
C16	0.0629 (17)	0.0500 (15)	0.0823 (19)	−0.0071 (14)	0.0130 (15)	−0.0228 (14)
C17	0.0239 (10)	0.0388 (12)	0.0344 (11)	−0.0050 (9)	−0.0025 (9)	−0.0015 (9)
C18	0.0357 (12)	0.0439 (13)	0.0361 (12)	−0.0050 (10)	−0.0057 (10)	0.0028 (10)
C19	0.0500 (14)	0.0397 (13)	0.0412 (13)	−0.0080 (11)	−0.0017 (11)	0.0032 (10)
C20	0.0754 (18)	0.0564 (16)	0.0459 (15)	−0.0174 (14)	−0.0037 (14)	0.0116 (12)
C21	0.0273 (11)	0.0377 (12)	0.0327 (11)	0.0011 (9)	−0.0103 (9)	0.0000 (9)
C22	0.0341 (12)	0.0475 (13)	0.0370 (12)	−0.0077 (11)	−0.0039 (10)	0.0035 (10)
C23	0.0481 (13)	0.0433 (13)	0.0341 (12)	−0.0077 (11)	−0.0071 (11)	0.0017 (10)
C24	0.0762 (18)	0.0606 (16)	0.0462 (15)	−0.0233 (15)	−0.0104 (14)	0.0130 (13)
C25	0.0332 (12)	0.0395 (12)	0.0326 (11)	−0.0081 (10)	−0.0082 (9)	−0.0041 (9)
C26	0.0433 (13)	0.0457 (14)	0.0456 (14)	−0.0005 (11)	−0.0070 (11)	−0.0096 (11)
C27	0.0691 (18)	0.0645 (17)	0.0581 (16)	0.0015 (15)	−0.0169 (14)	−0.0282 (14)
C28	0.112 (3)	0.081 (2)	0.082 (2)	0.0331 (19)	−0.040 (2)	−0.0439 (17)

### Geometric parameters (Å, º)

**Table:**

Fe1—O1	1.8608 (13)	C12—H12B	0.9800
Fe1—O2	1.8591 (13)	C12—H12C	0.9800
Fe1—O3	1.8515 (14)	N1—C21	1.515 (2)
Fe1—O4	1.8583 (13)	N1—C17	1.518 (2)
Si1—O1	1.5993 (14)	N1—C13	1.518 (2)
Si1—C3	1.857 (2)	N1—C25	1.520 (2)
Si1—C1	1.864 (2)	C13—C14	1.514 (3)
Si1—C2	1.869 (2)	C13—H13A	0.9900
Si2—O2	1.6071 (14)	C13—H13B	0.9900
Si2—C4	1.847 (2)	C14—C15	1.507 (3)
Si2—C6	1.853 (2)	C14—H14A	0.9900
Si2—C5	1.870 (2)	C14—H14B	0.9900
Si3—O3	1.6031 (15)	C15—C16	1.515 (3)
Si3—C9	1.855 (2)	C15—H15A	0.9900
Si3—C8	1.862 (2)	C15—H15B	0.9900
Si3—C7	1.864 (2)	C16—H16A	0.9800
Si4—O4	1.6143 (13)	C16—H16B	0.9800
Si4—C10	1.859 (2)	C16—H16C	0.9800
Si4—C11	1.863 (2)	C17—C18	1.520 (2)
Si4—C12	1.871 (2)	C17—H17A	0.9900
C1—H1A	0.9800	C17—H17B	0.9900
C1—H1B	0.9800	C18—C19	1.510 (3)
C1—H1C	0.9800	C18—H18A	0.9900
C2—H2A	0.9800	C18—H18B	0.9900
C2—H2B	0.9800	C19—C20	1.511 (3)
C2—H2C	0.9800	C19—H19A	0.9900
C3—H3A	0.9800	C19—H19B	0.9900
C3—H3B	0.9800	C20—H20A	0.9800
C3—H3C	0.9800	C20—H20B	0.9800
C4—H4A	0.9800	C20—H20C	0.9800
C4—H4B	0.9800	C21—C22	1.519 (2)
C4—H4C	0.9800	C21—H21A	0.9900
C5—H5A	0.9800	C21—H21B	0.9900
C5—H5B	0.9800	C22—C23	1.516 (2)
C5—H5C	0.9800	C22—H22A	0.9900
C6—H6A	0.9800	C22—H22B	0.9900
C6—H6B	0.9800	C23—C24	1.524 (3)
C6—H6C	0.9800	C23—H23A	0.9900
C7—H7A	0.9800	C23—H23B	0.9900
C7—H7B	0.9800	C24—H24A	0.9800
C7—H7C	0.9800	C24—H24B	0.9800
C8—H8A	0.9800	C24—H24C	0.9800
C8—H8B	0.9800	C25—C26	1.519 (3)
C8—H8C	0.9800	C25—H25A	0.9900
C9—H9A	0.9800	C25—H25B	0.9900
C9—H9B	0.9800	C26—C27	1.520 (3)
C9—H9C	0.9800	C26—H26A	0.9900
C10—H10A	0.9800	C26—H26B	0.9900
C10—H10B	0.9800	C27—C28	1.510 (3)
C10—H10C	0.9800	C27—H27A	0.9900
C11—H11A	0.9800	C27—H27B	0.9900
C11—H11B	0.9800	C28—H28A	0.9800
C11—H11C	0.9800	C28—H28B	0.9800
C12—H12A	0.9800	C28—H28C	0.9800
			
O3—Fe1—O4	112.58 (6)	H12A—C12—H12C	109.5
O3—Fe1—O2	105.76 (6)	H12B—C12—H12C	109.5
O4—Fe1—O2	111.55 (6)	C21—N1—C17	105.74 (13)
O3—Fe1—O1	112.53 (7)	C21—N1—C13	111.41 (14)
O4—Fe1—O1	105.17 (6)	C17—N1—C13	111.40 (14)
O2—Fe1—O1	109.33 (6)	C21—N1—C25	111.07 (14)
O1—Si1—C3	111.45 (9)	C17—N1—C25	111.13 (15)
O1—Si1—C1	109.84 (10)	C13—N1—C25	106.18 (13)
C3—Si1—C1	108.97 (13)	C14—C13—N1	116.35 (15)
O1—Si1—C2	110.30 (11)	C14—C13—H13A	108.2
C3—Si1—C2	107.99 (12)	N1—C13—H13A	108.2
C1—Si1—C2	108.20 (12)	C14—C13—H13B	108.2
O2—Si2—C4	111.65 (10)	N1—C13—H13B	108.2
O2—Si2—C6	109.81 (10)	H13A—C13—H13B	107.4
C4—Si2—C6	109.42 (14)	C15—C14—C13	111.60 (16)
O2—Si2—C5	110.26 (11)	C15—C14—H14A	109.3
C4—Si2—C5	107.23 (12)	C13—C14—H14A	109.3
C6—Si2—C5	108.38 (13)	C15—C14—H14B	109.3
O3—Si3—C9	111.92 (10)	C13—C14—H14B	109.3
O3—Si3—C8	110.69 (9)	H14A—C14—H14B	108.0
C9—Si3—C8	107.22 (11)	C14—C15—C16	113.43 (18)
O3—Si3—C7	110.32 (10)	C14—C15—H15A	108.9
C9—Si3—C7	108.60 (11)	C16—C15—H15A	108.9
C8—Si3—C7	107.95 (11)	C14—C15—H15B	108.9
O4—Si4—C10	110.89 (9)	C16—C15—H15B	108.9
O4—Si4—C11	109.98 (9)	H15A—C15—H15B	107.7
C10—Si4—C11	109.09 (10)	C15—C16—H16A	109.5
O4—Si4—C12	110.34 (9)	C15—C16—H16B	109.5
C10—Si4—C12	108.16 (11)	H16A—C16—H16B	109.5
C11—Si4—C12	108.32 (11)	C15—C16—H16C	109.5
Si1—O1—Fe1	137.69 (9)	H16A—C16—H16C	109.5
Si2—O2—Fe1	137.57 (8)	H16B—C16—H16C	109.5
Si3—O3—Fe1	151.05 (10)	N1—C17—C18	115.47 (14)
Si4—O4—Fe1	139.01 (8)	N1—C17—H17A	108.4
Si1—C1—H1A	109.5	C18—C17—H17A	108.4
Si1—C1—H1B	109.5	N1—C17—H17B	108.4
H1A—C1—H1B	109.5	C18—C17—H17B	108.4
Si1—C1—H1C	109.5	H17A—C17—H17B	107.5
H1A—C1—H1C	109.5	C19—C18—C17	111.02 (15)
H1B—C1—H1C	109.5	C19—C18—H18A	109.4
Si1—C2—H2A	109.5	C17—C18—H18A	109.4
Si1—C2—H2B	109.5	C19—C18—H18B	109.4
H2A—C2—H2B	109.5	C17—C18—H18B	109.4
Si1—C2—H2C	109.5	H18A—C18—H18B	108.0
H2A—C2—H2C	109.5	C18—C19—C20	111.97 (17)
H2B—C2—H2C	109.5	C18—C19—H19A	109.2
Si1—C3—H3A	109.5	C20—C19—H19A	109.2
Si1—C3—H3B	109.5	C18—C19—H19B	109.2
H3A—C3—H3B	109.5	C20—C19—H19B	109.2
Si1—C3—H3C	109.5	H19A—C19—H19B	107.9
H3A—C3—H3C	109.5	C19—C20—H20A	109.5
H3B—C3—H3C	109.5	C19—C20—H20B	109.5
Si2—C4—H4A	109.5	H20A—C20—H20B	109.5
Si2—C4—H4B	109.5	C19—C20—H20C	109.5
H4A—C4—H4B	109.5	H20A—C20—H20C	109.5
Si2—C4—H4C	109.5	H20B—C20—H20C	109.5
H4A—C4—H4C	109.5	N1—C21—C22	116.20 (14)
H4B—C4—H4C	109.5	N1—C21—H21A	108.2
Si2—C5—H5A	109.5	C22—C21—H21A	108.2
Si2—C5—H5B	109.5	N1—C21—H21B	108.2
H5A—C5—H5B	109.5	C22—C21—H21B	108.2
Si2—C5—H5C	109.5	H21A—C21—H21B	107.4
H5A—C5—H5C	109.5	C23—C22—C21	110.69 (15)
H5B—C5—H5C	109.5	C23—C22—H22A	109.5
Si2—C6—H6A	109.5	C21—C22—H22A	109.5
Si2—C6—H6B	109.5	C23—C22—H22B	109.5
H6A—C6—H6B	109.5	C21—C22—H22B	109.5
Si2—C6—H6C	109.5	H22A—C22—H22B	108.1
H6A—C6—H6C	109.5	C22—C23—C24	111.91 (17)
H6B—C6—H6C	109.5	C22—C23—H23A	109.2
Si3—C7—H7A	109.5	C24—C23—H23A	109.2
Si3—C7—H7B	109.5	C22—C23—H23B	109.2
H7A—C7—H7B	109.5	C24—C23—H23B	109.2
Si3—C7—H7C	109.5	H23A—C23—H23B	107.9
H7A—C7—H7C	109.5	C23—C24—H24A	109.5
H7B—C7—H7C	109.5	C23—C24—H24B	109.5
Si3—C8—H8A	109.5	H24A—C24—H24B	109.5
Si3—C8—H8B	109.5	C23—C24—H24C	109.5
H8A—C8—H8B	109.5	H24A—C24—H24C	109.5
Si3—C8—H8C	109.5	H24B—C24—H24C	109.5
H8A—C8—H8C	109.5	C26—C25—N1	115.46 (15)
H8B—C8—H8C	109.5	C26—C25—H25A	108.4
Si3—C9—H9A	109.5	N1—C25—H25A	108.4
Si3—C9—H9B	109.5	C26—C25—H25B	108.4
H9A—C9—H9B	109.5	N1—C25—H25B	108.4
Si3—C9—H9C	109.5	H25A—C25—H25B	107.5
H9A—C9—H9C	109.5	C25—C26—C27	111.05 (17)
H9B—C9—H9C	109.5	C25—C26—H26A	109.4
Si4—C10—H10A	109.5	C27—C26—H26A	109.4
Si4—C10—H10B	109.5	C25—C26—H26B	109.4
H10A—C10—H10B	109.5	C27—C26—H26B	109.4
Si4—C10—H10C	109.5	H26A—C26—H26B	108.0
H10A—C10—H10C	109.5	C28—C27—C26	112.4 (2)
H10B—C10—H10C	109.5	C28—C27—H27A	109.1
Si4—C11—H11A	109.5	C26—C27—H27A	109.1
Si4—C11—H11B	109.5	C28—C27—H27B	109.1
H11A—C11—H11B	109.5	C26—C27—H27B	109.1
Si4—C11—H11C	109.5	H27A—C27—H27B	107.9
H11A—C11—H11C	109.5	C27—C28—H28A	109.5
H11B—C11—H11C	109.5	C27—C28—H28B	109.5
Si4—C12—H12A	109.5	H28A—C28—H28B	109.5
Si4—C12—H12B	109.5	C27—C28—H28C	109.5
H12A—C12—H12B	109.5	H28A—C28—H28C	109.5
Si4—C12—H12C	109.5	H28B—C28—H28C	109.5
			
C3—Si1—O1—Fe1	−3.07 (19)	O2—Fe1—O4—Si4	66.36 (15)
C1—Si1—O1—Fe1	−123.94 (15)	O1—Fe1—O4—Si4	−175.23 (13)
C2—Si1—O1—Fe1	116.88 (16)	C21—N1—C13—C14	63.1 (2)
O3—Fe1—O1—Si1	55.77 (16)	C17—N1—C13—C14	−54.7 (2)
O4—Fe1—O1—Si1	178.67 (13)	C25—N1—C13—C14	−175.85 (16)
O2—Fe1—O1—Si1	−61.44 (16)	N1—C13—C14—C15	−177.98 (16)
C4—Si2—O2—Fe1	−7.49 (19)	C13—C14—C15—C16	−174.84 (18)
C6—Si2—O2—Fe1	−129.04 (15)	C21—N1—C17—C18	174.73 (16)
C5—Si2—O2—Fe1	111.60 (15)	C13—N1—C17—C18	−64.1 (2)
O3—Fe1—O2—Si2	178.46 (13)	C25—N1—C17—C18	54.1 (2)
O4—Fe1—O2—Si2	55.75 (15)	N1—C17—C18—C19	173.01 (17)
O1—Fe1—O2—Si2	−60.14 (15)	C17—C18—C19—C20	174.14 (18)
C9—Si3—O3—Fe1	12.8 (2)	C17—N1—C21—C22	−176.67 (16)
C8—Si3—O3—Fe1	132.34 (18)	C13—N1—C21—C22	62.1 (2)
C7—Si3—O3—Fe1	−108.2 (2)	C25—N1—C21—C22	−56.0 (2)
O4—Fe1—O3—Si3	−48.4 (2)	N1—C21—C22—C23	177.55 (16)
O2—Fe1—O3—Si3	−170.41 (18)	C21—C22—C23—C24	179.85 (18)
O1—Fe1—O3—Si3	70.3 (2)	C21—N1—C25—C26	−58.1 (2)
C10—Si4—O4—Fe1	7.56 (17)	C17—N1—C25—C26	59.3 (2)
C11—Si4—O4—Fe1	128.31 (14)	C13—N1—C25—C26	−179.38 (16)
C12—Si4—O4—Fe1	−112.26 (15)	N1—C25—C26—C27	−177.93 (17)
O3—Fe1—O4—Si4	−52.36 (15)	C25—C26—C27—C28	176.0 (2)
